# Knowledge, Attitude and Practice Regarding HIV/AIDS Among Antenatal Women Attending a Tertiary Care Center in Western India

**DOI:** 10.7759/cureus.90932

**Published:** 2025-08-25

**Authors:** Aruna Verma, Ritu Bharti, Monika Kashyap

**Affiliations:** 1 Obstetrics and Gynaecology, Lala Lajpat Rai Memorial (LLRM) Medical College, Meerut, IND

**Keywords:** antenatal women, antiretroviral therapy (art), hiv, hiv awareness, knowledge attitude practice (kap), mother-to-child transmission (mtct), pregnancy, prevention of mother-to-child transmission (pmtct)

## Abstract

Introduction: HIV infection remains a major global health issue, particularly affecting women and children through mother-to-child transmission (MTCT). In India, despite national programs and improved screening efforts, gaps persist in awareness, attitude, and practices among antenatal women regarding HIV transmission and its prevention. Identifying these gaps is essential for improving prevention of mother-to-child transmission (PMTCT) outcomes.

Aim & objectives: The aim of this study was to assess whether antenatal women attending a tertiary care center possess adequate knowledge, attitude, and preventive practices related to HIV. The specific objectives were to evaluate their understanding of HIV transmission and MTCT and determine key influencing factors.

Methodology: This hospital-based cross-sectional study was conducted in the Department of Obstetrics and Gynecology at LLRM Medical College, Meerut, from June 2023 to May 2024. A total of 500 antenatal women were enrolled using systematic sampling. Data was collected through a structured questionnaire and analyzed using IBM SPSS Statistics for Windows, Version 24 (Released 2015; IBM Corp., Armonk, New York, United States), applying univariate and multivariate logistic regression.

Results: The study found that 77.0% of women identified sexual transmission correctly, 64.0% recognized MTCT, and 75.0% acknowledged condom use as a preventive method. However, awareness about antiretroviral therapy during pregnancy (55.0%), breastfeeding risk (39.0%), and elective C-section (29.0%) was suboptimal. This highlights the need for targeted educational interventions to improve PMTCT awareness and practice.

Conclusion: The study reveals that while antenatal women in the study had a good level of awareness about HIV transmission routes and preventive measures, significant gaps in knowledge remain, particularly regarding MTCT and breastfeeding as a transmission route.

## Introduction

Human immunodeficiency virus (HIV) infection gradually weakens the body's immune system and, without adequate treatment, progresses to acquired immunodeficiency syndrome (AIDS). Though a cure is not currently available, antiretroviral therapy (ART) enables individuals to lead long, healthy lives while significantly reducing the risk of transmission [1]. The origin of HIV is traced back to chimpanzees in Central Africa, where it crossed into humans, possibly in the late 1800s through hunting activities. The virus spread through Africa and globally, with the first reported U.S. cases in the 1970s [2]. After infection, many individuals experience flu-like symptoms within 2-4 weeks, while others may remain asymptomatic for years. A confirmatory diagnosis can only be made through testing. The disease burden remains high, especially in low- and middle-income nations [3,4].

India ranks third globally in terms of the number of people living with HIV. The HIV prevalence among adults aged 15-49 years has significantly declined from 0.55% in 2000 to 0.32% in 2010 and further to 0.21% by 2021. Northeastern states like Mizoram (2.70%), Nagaland (1.36%), and Manipur (1.05%) report the highest prevalence, followed by Andhra Pradesh (0.67%), Telangana (0.47%), and Karnataka (0.46%) [5]. The estimated number of PLHIV in India was 2.4 million (1.93-3.04 million) in 2009, of which 39% are women, children under 15 years of age account for 4.4% of all infections, while people aged 15-49 years account for 82.4% of all infections [6]. Out of many, mother-to-child transmission (MTCT) is the major route of infection in children, which may occur during the antepartum and intrapartum period, or during breastfeeding [7].

Another positive development in this direction is the increased coverage from 0.8 million (in 2004) to 8.83 million (in 2013) in the number of pregnant women who are tested annually for HIV infection [8]. The National AIDS Control Program (NACP) and the National Rural Health Mission (NRHM) introduced universal HIV screening during routine ANC visits in 2010 to link HIV-positive pregnant women with appropriate prevention and treatment services [9]. The National Family Health Survey (NFHS-5) highlights that educating mothers about MTCT and ensuring access to ART are key to reducing neonatal infections [10].

Universal screening of all pregnant women, regardless of risk, combined with pre- and post-test counseling, is now considered more effective and cost-efficient. Without intervention, the risk of mother-to-child HIV transmission ranges from 20% to 50%: 5-10% during pregnancy, 10-20% during delivery, and 5-20% through breastfeeding. With the implementation of the prevention of mother-to-child transmission (PMTCT) program, this risk has dropped to below 2% [11].

In India, older models such as Voluntary Counseling and Testing Centers (VCTCs) and standalone PPTCT facilities have been restructured into Integrated Counseling and Testing Centers (ICTCs), offering consolidated services under one roof. Improving PMTCT practices contributes directly to the Millennium Development Goals (MDGs), particularly in reducing maternal and infant mortality and enhancing national health indicators [12]. PMTCT interventions include antenatal HIV testing and counseling, ART during pregnancy, safe delivery practices, and counseling on infant feeding. Nevertheless, challenges persist, such as social stigma, inadequate knowledge, and poor access in rural areas, which hinder the effectiveness of MTCT prevention [13].

Despite the availability of services, NFHS data reveal that many pregnant women in India still lack adequate knowledge, practice, and attitude regarding HIV transmission during childbirth and breastfeeding. Misconceptions, fear of discrimination, and lack of awareness significantly limit PMTCT uptake [14]. A mother’s level of understanding and attitude toward HIV directly influences her willingness to participate in prevention programs. Women with good knowledge and positive attitudes are more likely to attend counseling and testing sessions and adhere to treatment protocols compared to those lacking awareness [15,16].

Against this backdrop, the current study was planned to assess the knowledge, attitudes, and practices (KAP) of antenatal women regarding HIV and PMTCT. By correlating these factors with sociodemographic characteristics, the study will help to identify educational gaps and barriers to PMTCT utilization. Ultimately, such research will play a vital role in curbing mother-to-child HIV transmission and contribute to achieving the long-term public health goal of eliminating AIDS as a threat.

## Materials and methods

The study was conducted in the Department of Obstetrics and Gynecology, LLRM Medical College, Meerut. It was a hospital-based, cross-sectional, questionnaire-based study and started after obtaining permission from the institutional ethical committee (No./SC-1/2025/2923). All pregnant women attending the ANC OPD at SVBP Hospital, Meerut, who gave written and informed consent were included. Women who did not provide consent and were severely ill (those who could not communicate) were excluded from the study.

The sample size was calculated based on a previous study [17] that reported the prevalence of poor knowledge regarding mother-to-child transmission of HIV/AIDS as 33.33%. Using a 95% level of confidence and an error rate, typically set at 0.05, the total number of participants was determined to be 340. The formula used for calculation was:

n = Z²P(1 - P)/d²

Where: n = sample size, Z = Z statistic for the level of confidence; for a 95%

confidence level, the Z value is 1.96, P = expected prevalence or proportion (if

33%, P = 0.33), d = precision (if 5%, d = 0.05).

The calculation was as follows: n = 1.96 × 1.96 × 0.33 × 0.67 / 0.05²

n = 339.75

However, a total of 500 eligible participants were ultimately enrolled after applying the inclusion criteria.

The source of data for the study was the structured questionnaire (SQF) form filled out by either pregnant women or with the help of healthcare workers/doctors during their first antenatal visit, as per information provided by the antenatal women (included in the appendix). A systematic sampling technique was used, where every alternate pregnant woman registered for ANC visits was voluntarily included in the study after a careful explanation about the study, and their consent was obtained. The SQF contained all demographic parameters including age, address, religion, educational status, occupation, and total monthly income.

After the data collection, each questionnaire was thoroughly reviewed for consistency and completeness by the data collector and supervisor. The data were then reentered into Epi Info Version 3.5 and analyzed using IBM SPSS Statistics for Windows, Version 24 (Released 2015; IBM Corp., Armonk, New York, United States). Any data anomalies and outliers were cleaned by reviewing descriptive statistics (frequency). Frequencies and proportions were first calculated to describe the respondents’ knowledge, attitudinal response, and practice towards HIV/AIDS among antenatal women. Univariate logistic regression analysis was performed to determine the impact of select knowledge and attitude indicators on the respondents' risk perception and preventive practice adopted in light of HIV/AIDS among antenatal women. Multivariate logistic regression analysis was applied to investigate socio-demographic factors that may be associated with risk perception. Adjusted odds ratios corresponding to 95% confidence intervals (Cls) were obtained using multiple logistic regression analysis.

## Results

A total of 500 cases were enrolled after fulfilling the inclusion criteria. Out of the total study participants, 214 (42.8%) were in the 18-25 years age group, 176 (35.2%) were in the 26-30 years age group, 82 (16.4%) were in the 31-35 years age group, and 28 (5.6%) were in the >35 years age group. Out of the total enrolled cases, 16.0% were illiterate, 29.0% had and 11.0% were graduates. out of the total study participants, 6.0% belonged to the Upper Class (I), 17.0% to the Upper Middle Class (II), 35.0% to the Lower Middle Class (III), 39.0% to the Upper Lower Class (IV), and 3.0% to the Lower Class (V).

**Figure 1:**
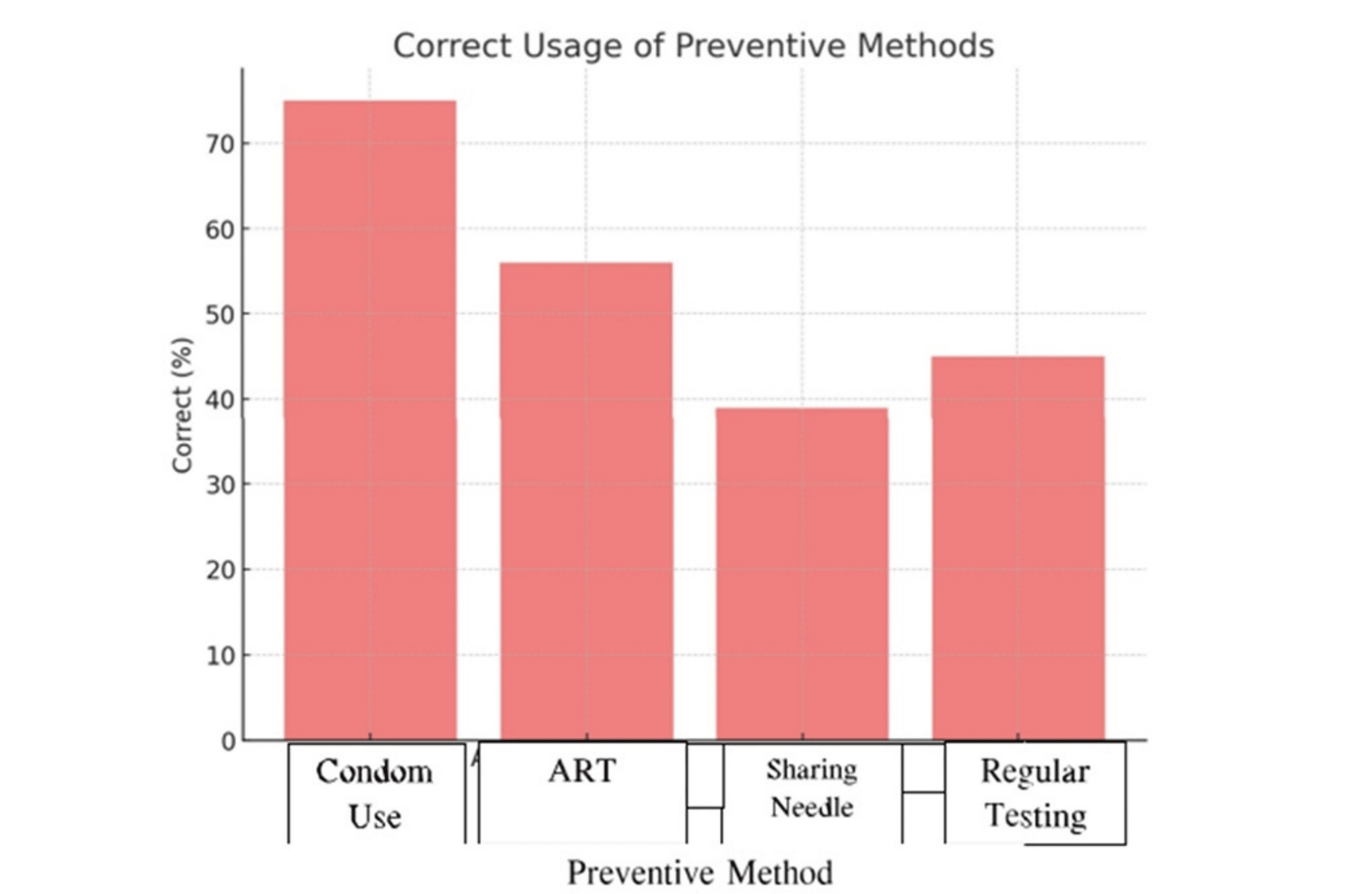
Awareness of HIV Transmission Routes HIV: Human Immunodeficiency Virus; ART: Antiretroviral therapy

Figure 1 illustrates that out of the total study participants, 375 (75.0%) reported correct condom use. Additionally, 280 (56.0%) participants reported correct use of ART therapy, while 195 (39.0%) correctly followed the practice of avoiding sharing needles. Furthermore, 225 (45.0%) participants adhered to regular testing.

**Figure 2:**
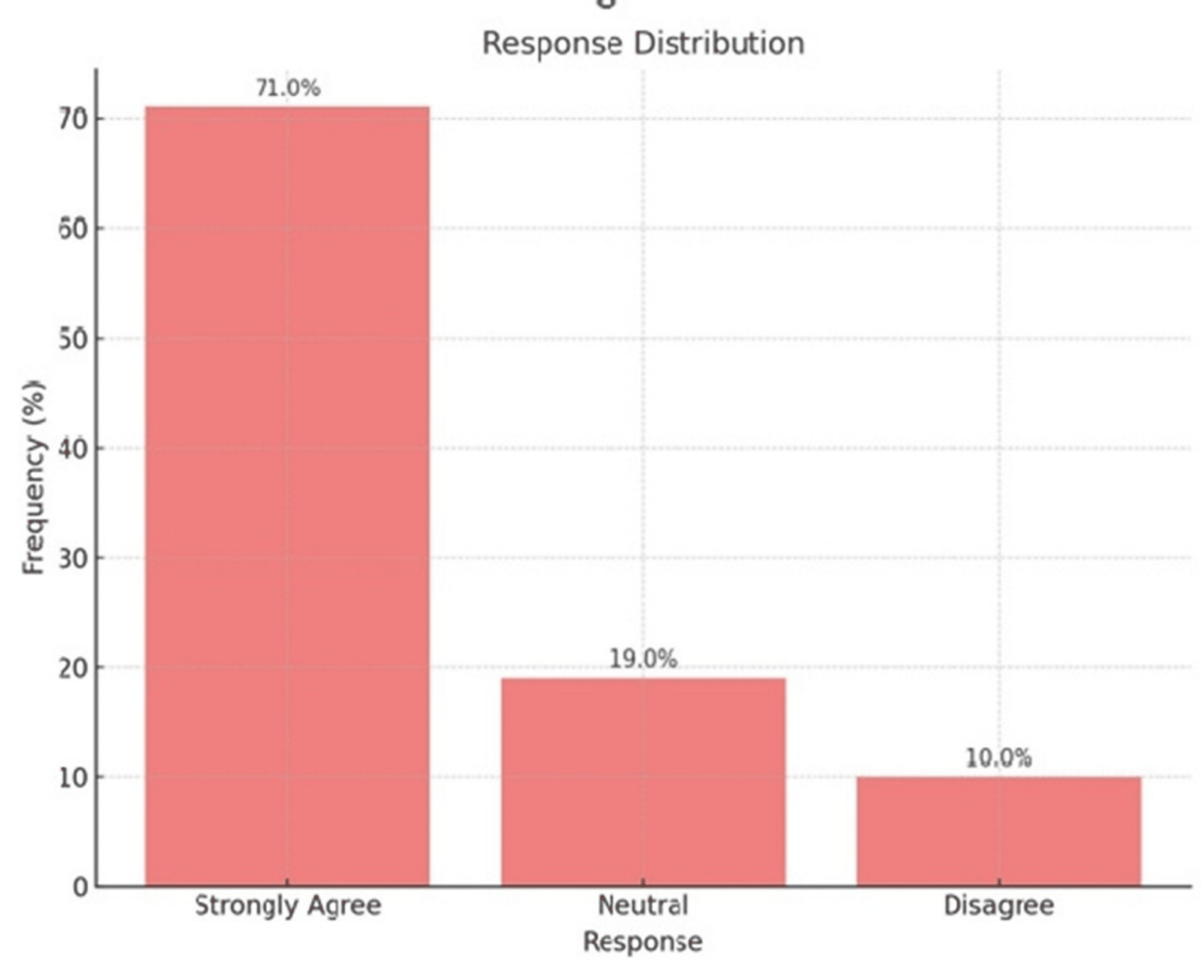
Importance of HIV Testing in Pregnancy HIV: Human Immunodeficiency Virus

Figure 2 shows that out of the total study participants, 355 (71.0%) strongly agreed, 95 (19.0%) were neutral, and 50 (10.0%) to disagree.

**Figure 3:**
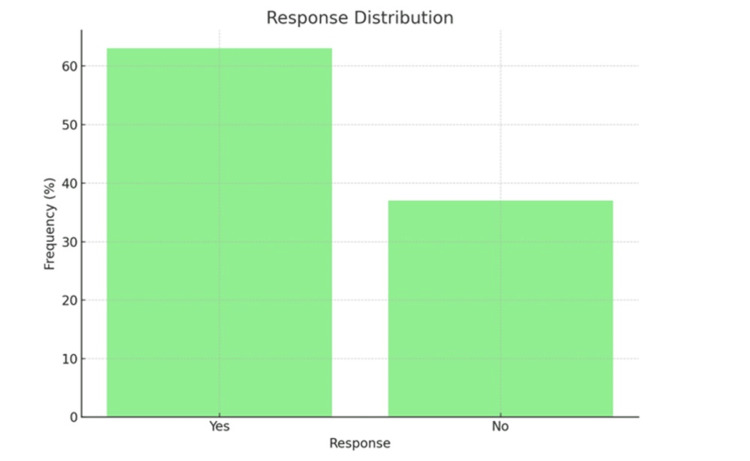
Willingness to Disclose HIV Status HIV: Human Immunodeficiency Virus

Figure 3 illustrates that out of the total study participants, 315 (63.0%) responded with "Yes" and 185 (37.0%) responded with "No”, if they are asked to disclose their HIV status.

**Figure 4:**
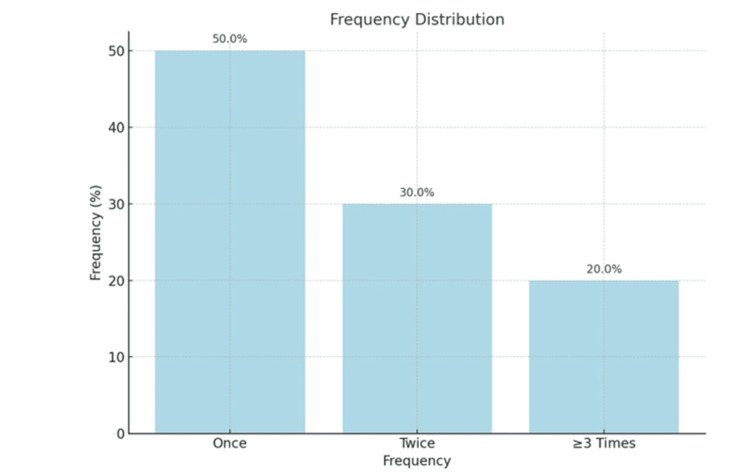
Frequency of HIV Testing HIV: Human Immunodeficiency Virus

Figure 4 depicts that out of total study participants, 250 (50.0%) reported having the experience once of HIV testing, 150 (30.0%) reported it twice, and 100 (20.0%) reported it three or more times.

**Figure 5:**
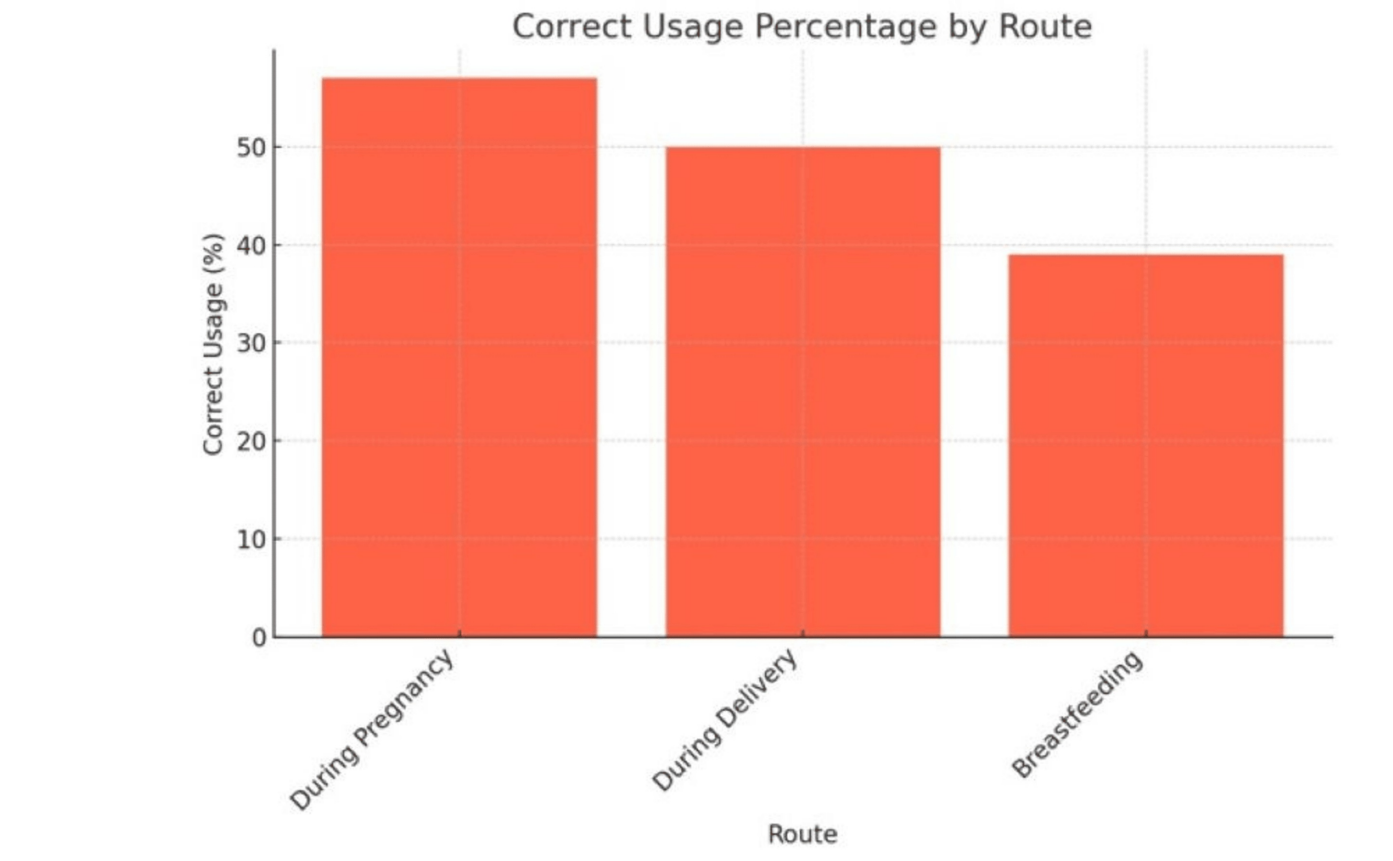
Awareness of MTCT Routes MTCT: Mother-to-Child Transmission

Figure 5 illustrates that out of the total study participants, 285 (57.0%) correctly identified pregnancy as a route of mother-to-child transmission of HIV, 250 (50.0%) identified delivery, and 195 (39.0%) identified breastfeeding.

**Figure 6:**
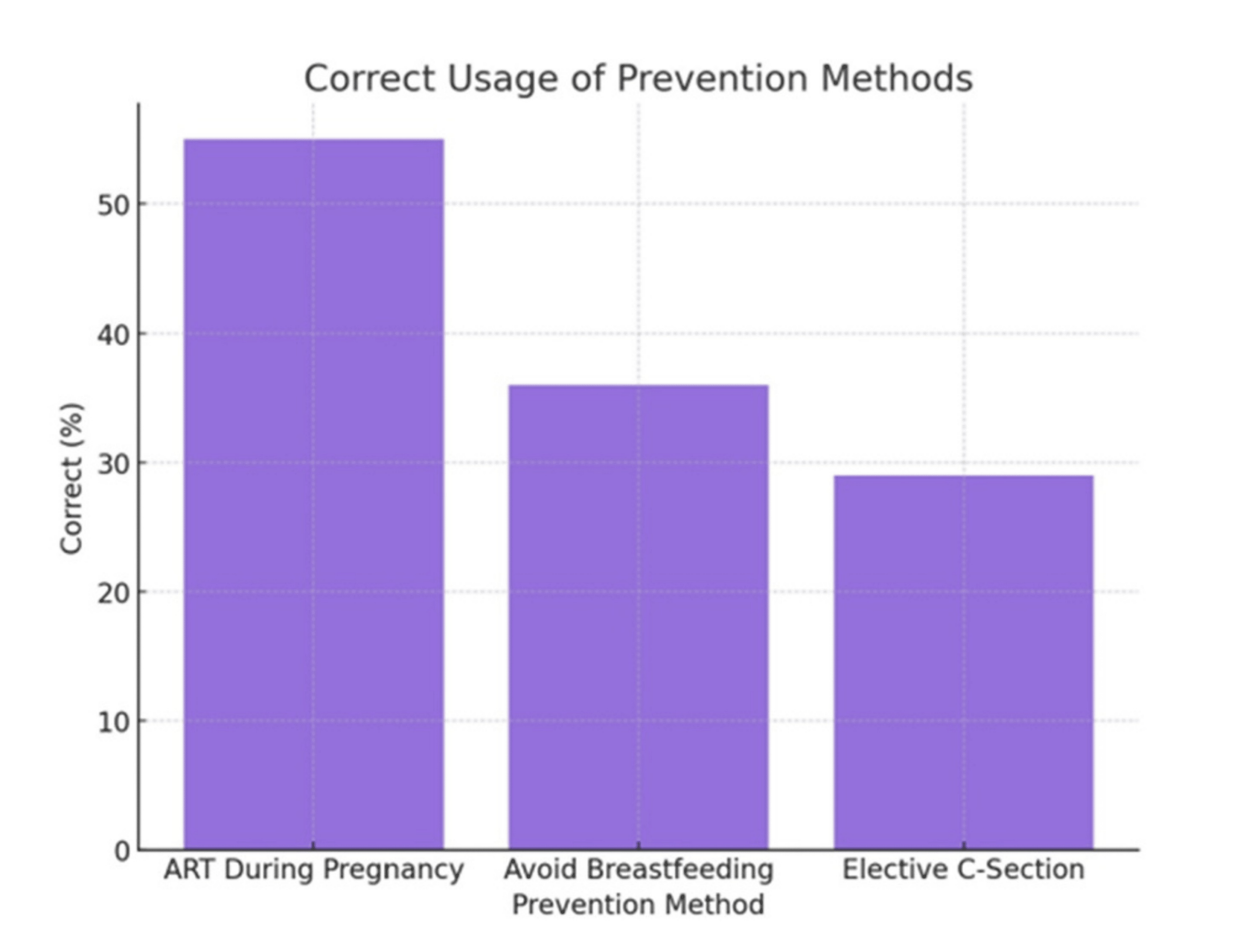
Knowledge of MTCT Prevention MTCT: Mother-to-Child Transmission

Figure 6 shows that out of total study participants, 275 (55.0%) correctly identified ART during pregnancy as a preventive method. Additionally, 180 (36.0%) correctly identified avoiding breastfeeding as a preventive method, and 145 (29.0%) correctly identified elective C-section as a preventive method for MTCT of HIV.

**Figure 7:**
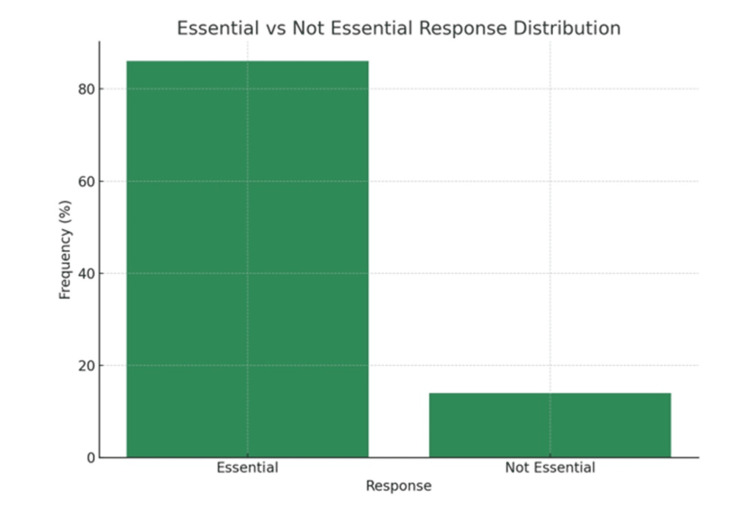
Attitude Toward ANC Follow-Up ANC: Antenatal Care

Figure 7 illustrates that among the total 500 study participants,430 (86.0%) considered regular ANC follow-up essential for ensuring maternal and fetal health, whereas 70 (14.0%) did not perceive it as necessary. These findings reflect a predominantly positive attitude toward ANC services, with a small proportion requiring targeted awareness efforts.

Table 1 illustrates that out of the total study participants, education level showed a correlation coefficient of 0.42 with a p-value of <0.001, indicating a significant positive relationship. Socioeconomic status had a correlation coefficient of 0.35 with a p-value of 0.002, also showing a significant positive correlation. Age exhibited a correlation coefficient of 0.18 with a p-value of 0.064, which was not statistically significant. ANC attendance had a correlation coefficient of 0.28 with a p-value of 0.008, demonstrating a significant positive correlation.

**Table 1:** Correlation Between Sociodemographic Factors and HIV Knowledge Scores HIV: Human Immunodeficiency Virus

Variable	Correlation Coefficient (r)	p- value
Education Level	0.42	<0.001
Socioeconomic Status	0.35	0.002
Age	0.18	0.064
ANC Attendance	0.28	0.008
Spearman’s rank correlation significant at p<0.05		

Table 2 illustrates that out of the total study participants, there was a positive correlation between knowledge and testing practice with a correlation coefficient of 0.38 (p<0.001). A stronger positive correlation was found between knowledge and PMTCT support with a correlation coefficient of 0.45 (p<0.001). Additionally, attitude was positively correlated with ANC follow-up, showing a correlation coefficient of 0.32 (p=0.003). All correlations were statistically significant with a p-value of less than 0.05.

**Table 2:** Correlation Between HIV Knowledge and Attitude/Practice HIV: Human Immunodeficiency Virus

Variable	Correlation Coefficient (r)	p-value
Knowledge vs. Testing Practice	0.38	<0.001
Knowledge vs. PMTCT Support	0.45	<0.001
Attitude vs. ANC Follow-Up	0.32	0.003
Pearson’s correlation significant at p<0.05		

Table 3 shows that out of the total study participants, 250 (50.0%) had poor knowledge with a score range of 0-3, 175 (35.0%) had average knowledge with a score range of 4-6, and 75 (15.0%) had good knowledge with a score range of 7-10.

**Table 3:** Grading of HIV Knowledge (Score: 0–10) HIV: Human Immunodeficiency Virus

Category	Score Range	Frequency (%)
Poor Knowledge	0–3	250(50.0%)
Average Knowledge	4–6	175(35.0%)
Good Knowledge	7–10	75 (15.0%)

Table 4 illustrates that out of the total study participants, 80 participants (16.0%) had a negative attitude, with a score range of 0-4, 220 participants (44.0%) had a neutral attitude, with a score range of 5-7, and 200 participants (40.0%) had a positive attitude, with a score range of 8-10.

**Table 4:** Attitude Grading Toward MTCT MTCT: Mother-to-Child Transmission

Category	Score Range	Frequency (%)
Negative Attitude	0–4	80 (16.0%)
Neutral Attitude	5–7	220 (44.0%)
Positive Attitude	8–10	200 (40.0%)

Table 5 illustrates that out of the total study participants, 150 (30.0%) had poor practice, 275 (55.0%) had moderate practice, and 75 (15.0%) had good practice.

**Table 5:** Practice Scoring for HIV Prevention HIV: Human Immunodeficiency Virus

Category	Score Range	Frequency (%)
Poor Practice	0–2	150 (30.0%)
Moderate Practice	3–5	275 (55.0%)
Good Practice	6–8	75 (15.0%)

## Discussion

The present study was conducted to assess the knowledge, attitude, and preventive practices regarding HIV infection among antenatal women attending a tertiary care center, with a particular focus on their understanding of MTCT. The findings of the present study revealed that the majority of participants belonged to the age group of 18-25 years (42.8%), followed by 26-30 years (35.2%). This age distribution aligns closely with the findings of Nayak et al. [18], who also reported that the majority of HIV- positive pregnant women were in the 20-30-year age group. Such consistency in age-related vulnerability across studies emphasizes the need to target educational and preventive interventions specifically toward younger reproductive-age women.

Socioeconomic status played a critical role in shaping knowledge and practice outcomes. In this study, 39.0% of the participants belonged to the upper-lower class, and 35.0% to the lower-middle class. This is in accordance with the study by Antabe et al. [19]. They found that compared to those with no formal education, women with primary education (OR = 1.88, 95% CI = 1.04, 3.41) and secondary education or higher (OR = 2.61, 95% CI = 1.21, 5.62) were more likely to have adequate knowledge of MTCT of HIV.

Regarding educational status, this study found that 44.0% of participants had secondary education, while 29.0% had only primary education and 16.0% were illiterate. These results are echoed by Nayak et al. [18], who found that illiteracy was a significant risk factor for HIV infection among pregnant women, primarily due to reduced comprehension of prevention strategies and public health messaging. Similarly, Antabe et al. [19] also highlighted the inverse relationship between education level and HIV awareness, where women with lower educational attainment exhibited poorer knowledge about HIV transmission, including MTCT.

With respect to knowledge on HIV transmission, 77.0% of women in this study correctly identified sexual contact as a route of transmission, followed by 64.0% identifying mother-to-child transmission and 51.0% recognizing transmission via blood transfusion. Notably, only 42.0% identified breastfeeding as a potential mode of MTCT. These findings are consistent with Lucksom et al. [20], who reported that while a significant proportion of antenatal mothers were aware of unsafe sexual practices (90%) as a risk factor for HIV, only 2.66% recognized transmission through breast milk, indicating a critical knowledge gap in both studies. This similarity reinforces the need to enhance education about all routes of MTCT, especially breastfeeding, which remains under-recognized.

In terms of knowledge, 75.0% of participants in the current study correctly identified condom use as an effective preventive measure, while 56.0% acknowledged the role of ART, 39.0% recognized the importance of avoiding shared needles, and 45.0% reported regular HIV testing as a preventive strategy. These figures reflect moderate awareness, with condom use being the most commonly known preventive method. In contrast, Mukhtar et al. [21] reported that 85.8% of antenatal women were aware of sexual transmission, but only 50.8% knew about MTCT, indicating a similar trend where basic transmission knowledge was relatively higher than understanding of MTCT-related interventions. Similarly, Upadhyay et al. [22] noted unsatisfactory overall awareness, with only 54% of participants recognizing MTCT, mirroring the gap observed in the present study regarding ART and regular testing.

Regarding attitudes, the present study found that 71.0% of participants strongly agreed on the importance of HIV testing during pregnancy, which is consistent with findings from Yeshaneh et al. [23], who reported a positive attitude in 79% of antenatal women. 

The present study conducted at a tertiary care center revealed significant findings regarding the awareness of antenatal women about MTCT of HIV and its prevention. In this study, 57.0% of participants correctly identified pregnancy as a route of MTCT, 50.0% identified delivery, and only 39.0% recognized breastfeeding as a transmission route. These findings align partially with the results of Yeshaneh et al. [23], who reported that 38.9% of participants identified breastfeeding as a route of transmission, and overall, 72.2% demonstrated good knowledge of PMTCT. Compared to our results, their population showed slightly higher awareness levels, especially regarding multiple transmission routes.

When examining preventive methods for MTCT, 55.0% of participants in our study recognized ART during pregnancy as an effective measure, while only 36.0% and 29.0% identified avoiding breastfeeding and elective cesarean section, respectively. These results highlight moderate awareness among participants, like Upadhyay et al. [22]. They reported that 26% women were totally unaware of any entity like HIV. 44% participants did not know the most common way of spreading HIV. Only half of the subjects knew the correct preventive measures for HIV/AIDS. 54% knew about MTCT, but only 24% knew about its transmission through breast milk. In contrast, the population studied by Yeshaneh et al. [23] demonstrated comparatively higher awareness and adherence to preventive practices, including ART. The lower percentage in our cohort regarding knowledge of elective cesarean delivery as a preventive strategy indicates a continued need for targeted education interventions.

The study’s findings have important implications for HIV prevention strategies, particularly in the context of antenatal care. While most women in the study had adequate knowledge about HIV transmission routes and prevention methods, there were still significant gaps, especially in the area of breastfeeding as a route of transmission. This gap in knowledge could result in missed opportunities for preventing MTCT, as breastfeeding is a critical period during which transmission can occur. 

The main limitation of the study was that it was cross-sectional in nature, which only captured data at one point in time, limiting the ability to assess changes in knowledge, attitude, or practices over time. The results may not be generalizable to antenatal women attending primary or secondary care centers, as they may have different levels of education, access to healthcare, and attitudes toward HIV.

## Conclusions

The study reveals that while antenatal women in the study had a good level of awareness about HIV transmission routes and preventive measures, significant gaps in knowledge remain, particularly regarding MTCT and breastfeeding as a transmission route. The findings also indicate that education and socio-economic status play a critical role in shaping HIV knowledge and attitudes. Therefore, to enhance the effectiveness of PMTCT programs, it is essential to implement educational interventions that target women from low-education and low-income backgrounds. These interventions should focus on all aspects of MTCT prevention, including the importance of ART during pregnancy, elective cesarean delivery, and safe infant feeding practices. Strengthening PMTCT programs and ensuring that women have access to the necessary information and healthcare services will be key to reducing the transmission of HIV from mother to child and improving maternal and child health outcomes in India.

## Appendices

**Table 6:** Sructured Questionaire Credits: Aruna Verma PMTCT: Prevention of Mother-to-Child Transmission; HIV: Human Immunodeficiency Virus; MTCT: Mother-to-Child Transmission

1	Socio-demographic Characteristic		
Age			
Address		Rural	urban
Religion		Hindu	Muslim
Educational Status: Unable to read/Able to read/primary level/secondary level/College or University			
Occupation: Housewife/ Govt. Employee/Daily Labor/Farmer/Student			
Total Monthly Income: No income, <600/- ,600-1000, >1000			
2.	Knowledge of pregnant women about HIV infection		
Have you ever heard about HIV/AIDS –		Yes	No
Do you know the root of the transmission of HIV		Yes	No
(If yes) what way of HIV transmission do you know			
Sharing sharp measures		Yes	No
Unprotected Sexual Intercourse		Yes	No
Mother-to-child transmission		Yes	No
Do you know the above HIV preventive methods		Yes	No
(If yes, which preventive method do you know			
Barrier methods (condoms)		Yes	No
Special drugs		Yes	No
Others		Yes	No
(If yes) what is the advantage of these methods			
Protect the mother from being infected with HIV		Yes	No
Decrease the risk of having HIV HIV-infected baby		Yes	No
Prevent unwanted pregnancy & STIs		Yes	No
3.	Knowledge of pregnant women towards MTCT of HIV/AIDS and its prevention		
Is mother-to-child transmission of HIV/AIDS possible		Yes	No
(If yes) when MTCT of HIV occurs			
During Pregnancy		Yes	No
During Labor		Yes	No
During Breast Feeding		Yes	No
Don't know		Yes	No
Are you aware that MTCT of HIV can be prevented		Yes	No
Do you know about HIV Counseling and testing		Yes	No
Do you think testing has an advantage for PMTCT		Yes	No
If yes, then			
To know our status		Yes	No
To start ART for HIV positives		Yes	No
To protect others		Yes	No
To live a healthy life		Yes	No
If Medication (ART) given to the mother during the antenatal period reduce the MTCT of HIV		Yes	No
How can MTCT of HIV be prevented			
Durg (ART) therapy during pregnancy		Yes	No
Delivery by LSCS		Yes	No
Giving ART to newborns		Yes	No
No defined infant feeding, early infant test		Yes	No
Don't know		Yes	No
4.	Attitude of pregnant females towards MTCT of HIV and its prevention		
Is it important for pregnant women to get tested for HIV		Agree	Disagree
Does every pregnant female have to attend ANC follow-up		Agree	Disagree
If someone is infected, then she should not get pregnant		Agree	Disagree
Do you support the staging of PMTCT		Agree	Disagree
Using protective methods (Condom) during pregnancy and breastfeeding reduces MTCT.		Agree	Disagree
Obstetric care during labor and delivery reduces MTCT of HIV		Agree	Disagree
Women fear being tested for HIV/AIDS due to social isolation		Agree	Disagree
5.	Practice of pregnant mothers towards MTCT of HIV/AIDS and its prevention		
Have you ever been tested		Yes	No
Where did you get tested		Yes	No
Do you have testing practice every three months		Yes	No
Was any pretesting counselling done to you during HIV testing		Yes	No
Was any post testing counselling done to you during HIV testing		Yes	No
How many times were you tested for HIV (1/ 2/ 3/ >3			
Have you ever done anything to prevent MTCT as an individual		Yes	No
